# Integrated Human Immunodeficiency Virus Type 1 Sequence in J-Lat 10.6

**DOI:** 10.1128/MRA.00179-20

**Published:** 2020-04-30

**Authors:** Cheng-Han Chung, Anthony R. Mele, Alexander G. Allen, Robert Costello, Will Dampier, Michael R. Nonnemacher, Brian Wigdahl

**Affiliations:** a Department of Microbiology and Immunology, Drexel University College of Medicine, Philadelphia, Pennsylvania, USA; b Center for Molecular Virology and Translational Neuroscience, Institute for Molecular Medicine and Infectious Disease, Drexel University College of Medicine, Philadelphia, Pennsylvania, USA; c Center for Clinical and Translational Medicine, Institute for Molecular Medicine and Infectious Disease, Drexel University College of Medicine, Philadelphia, Pennsylvania, USA; d Sidney Kimmel Cancer Center, Thomas Jefferson University, Philadelphia, Pennsylvania, USA; Portland State University

## Abstract

The full length of HIV/R7/E^−^/GFP integrated in the J-Lat 10.6 cell line was sequenced in this study. The single copy of the integrated virus, including the breakpoints from the human chromosome to the provirus, was amplified by two separate PCRs. A 10,200-bp genome sequence was acquired, analyzed, and deposited in GenBank.

## ANNOUNCEMENT

The J-Lat 10.6 cell line is a subclone derived from Jurkat-based cells infected with a pseudotyped human immunodeficiency virus type 1 (HIV-1) (genus *Lentivirus*, family *Retroviridae*) strain, HIV/R7/E^−^/GFP (1, 2). The integrated HIV-1 copy in this cell line is located in the second intron of *SEC16A* (chromosome 9, position 136468579), providing a useful cell line for studying HIV-1 latency (3). In order to use J-Lat 10.6 for anti-HIV-1 gene editing and design strategies using the clustered regularly interspaced short palindromic repeats (CRISPR) system, it is necessary to have the full proviral DNA sequence (4–11). However, the full-length sequence of integrated HIV/R7/E^−^/GFP has not been reported. Here, we amplified two overlapping fragments and performed subsequent Sanger sequencing to acquire the whole genome of HIV/R7/E^−^/GFP.

Genomic DNA was isolated from J-Lat 10.6 cells utilizing the QIAamp DNA minikit (catalog number 51304; Qiagen) as described by the manufacturer. In order to determine the HIV-1 proviral sequence, the DNA was amplified as two fragments using newly designed primers (Table 1). The amplicon starting at the 5′ end of the provirus (5′ amplicon) was 8,999 bp and was amplified using primers based on the reported integration site (3); primers 10.6_up5LTR_F and eGFP-N (ReadyMade Primers, catalog number 51-01-05-05; Integrated DNA Technologies), directed to the N terminus of the gene for enhanced green fluorescent protein (eGFP), which replaces *nef* in this molecular clone, were used for the 5′ amplicon PCR. An adapted single-genome amplification protocol (12) using Platinum *Taq* polymerase (catalog number 10966026; Invitrogen) was implemented.

**TABLE 1:** Primers utilized for amplification and sequencing, including primer locations within the proviral genome and human genome, directionality, and sequences

Primer type and name	Sequence	Genome	Nucleotide position		Strand	Length (bp)
			Start	Stop		
Amplification						
10.6_up5LTR_F^*a*^	CGTACTGGCTGGAGTAATAGCT	hg38, chromosome 9	136468424	136468445	+	22
eGFP-N^*a*^	CGTCGCCGTCCAGCTCGACCAG	HIV	8776	8797	−	22
Frag-26-R-RC^*b*^	CTGGCTGTGGAAAGATACCT	HIV	7983	8002	+	20
10.6_down3LTR_R^*b*^	GAATGCCCATTGCTTTGGGAA	hg38, chromosome 9	136468587	136468607	−	21
Sequencing						
10.6_up5LTR_F	CGTACTGGCTGGAGTAATAGCT	hg38, chromosome 9	136468424	136468445	+	22
10.6_down3LTR_R	GAATGCCCATTGCTTTGGGAA	hg38, chromosome 9	136468587	136468607	−	21
275F	ACAGGGACCTGAAAGCGAAAG	HIV	646	666	+	21
275F-RC	CTTTCGCTTTCAGGTCCCTGT	HIV	646	666	−	21
Frag-22-R	GGGTAATTTTGGCTGACCTG	HIV	1168	1187	−	20
Frag-22-R-RC	CAGGTCAGCCAAAATTACCC	HIV	1168	1187	+	20
Frag-14-R	ATGTCACTTCCCCTTGGTTC	HIV	1477	1496	−	20
Frag-14-R-RC	GAACCAAGGGGAAGTGACAT	HIV	1477	1496	+	20
Frag-17-L	GGAAATGTGGAAAGGAAGGAC	HIV	2030	2050	+	21
Frag-17-L-RC	GTCCTTCCTTTCCACATTTCC	HIV	2030	2050	−	21
Frag-25-L	TGGATGGCCCAAAAGTTAAAC	HIV	2596	2616	+	21
Frag-33-R-RC	CACAGGGATGGAAAGGATCA	HIV	2998	3017	+	20
5INOut	ACTCCATCCTGATAAATGGACAG	HIV	3248	3270	+	23
Frag-10-L	ACAATTAACAGAGGCAGTGC	HIV	3647	3666	+	20
Frag-18-L	AGTGATTTTAACCTGCCACC	HIV	4299	4318	+	20
Frag-27-L	TGGTAGCAGTTCATGTAGCC	HIV	4450	4469	+	20
5AccOut	CGGGTTTATTACAGGGACARCARA	HIV	4899	4922	+	24
3InOut-RC	GGCAAGTAGACAGGATGAGGATT	HIV	5072	5094	+	23
Frag-37-L	AAAGCCACCTTTGCCTAGTG	HIV	5517	5536	+	20
Frag-36-R	CTGACTTCCTGGATGCTTCC	HIV	5862	5881	−	20
Frag-36-R-RC	GGAAGCATCCAGGAAGTCAG	HIV	5862	5881	+	20
Frag-05-L	AGCAGAAGACAGTGGCAATG	HIV	6208	6227	+	20
3AccOut-RC	ATAATRTYTGGGCCACACATGCC	HIV	6421	6443	+	23
Frag-09-L	TGGCAGTCTAGCAGAAGAAG	HIV	7010	7029	+	20
Frag-39-L	CAATGTATGCCCCTCCCATC	HIV	7522	7541	+	20
Frag-26-R-RC	CTGGCTGTGGAAAGATACCT	HIV	7965	7984	+	20
Frag-21-L	GAAGGTGGAGAGAGAGACAG	HIV	8430	8449	+	20
eGFP-N	CGTCGCCGTCCAGCTCGACCAG	HIV	8776	8797	−	22
eGFP-C	CATGGTCCTGCTGGAGTTCGTG	HIV	9517	9538	+	22

a Primers used for the 5′ amplicon (8,999 bp), with the following PCR conditions: 94°C for 2 min, (94°C for 30 s, 62°C for 30 s, and 68°C for 10 min) for 3 cycles, (94°C for 30 s, 58°C for 30 s, and 68°C for 10 min) for 3 cycles, (94°C for 30 s, 56°C for 30 s, and 68°C for 10 min) for 3 cycles, (94°C for 30 s, 53.5°C for 30 s, and 68°C for 10 min) for 21 cycles, and 68°C for 10 min.

b Primers used for the 3′ amplicon (2,140 bp), with the following PCR conditions: 98°C for 1 min, (98°C for 10 s, 68.8°C for 20 s, and 72°C for 1 min) for 30 cycles, and 72°C for 1 min.

The 3′ amplicon encompassed eGFP and the 3′ long terminal repeat (LTR) and was 2,140 bp. It was amplified using primers Frag-26-R-RC and 10.6_down3LTR_R, with the PCR conditions listed in Table 1. The PCR products were enzymatically purified utilizing ExoSAP-IT PCR product cleanup reagent (catalog number 78201.1.ML; Thermo Fisher Scientific). Sanger sequencing was performed by GENEWIZ, Inc. (South Plainfield, NJ), using Applied Biosystems BigDye version 3.1 and the primers listed in Table 1. The reactions were run on an Applied Biosystems 3730xl DNA analyzer.

Quality control of the sequence was performed by end trimming using average quality scores of >16 over 21 bp, followed by assembly with default settings in DNASTAR SeqMan (13). The entire HIV-1 proviral genome reported was 10,200 bp, with a GC content of 43.7%. Every nucleotide within HIV/R7/E^−^/GFP was sequenced at least twice for a high level of accuracy and was annotated by DNASTAR SeqBuilder for GenBank submission (13). Previously reported mutations in *vpr* and *env* and the resulting immature proteins were annotated in the GenBank file (2, 14). There is an insertion of thymine and adenine at nucleotide position 6548, which causes a frameshift of *env* and an early stop codon at amino acid residue 85. The *vpr* coding region has an insertion of thymine at nucleotide position 5919, which causes a frameshift of *vpr* and an early stop codon at Vpr amino acid residue 79.

### Data availability.

The accession number for the genome sequence of HIV/R7/E^−^/GFP and the flanking integration site is MN989412.
